# A Poly Resistor Based Time Domain CMOS Temperature Sensor with 9b SAR and Fine Delay Line

**DOI:** 10.3390/s20072053

**Published:** 2020-04-06

**Authors:** Zhiwei Xu, Sangjin Byun

**Affiliations:** Division of Electronics and Electrical Engineering, Dongguk University, Seoul 04620, Korea; xzwbruce@gmail.com

**Keywords:** temperature sensor, fine delay line, coarse delay line, SAR, time domain, CMOS integrated circuits

## Abstract

This paper presents a new type of time domain CMOS temperature sensor with a 9b successive approximation register (SAR) control logic and a fine delay line. We adopted an N-type poly resistor as the sensing element for temperature linearity. The chip was implemented in a standard 0.18 m 1P6M bulk CMOS process with general V_TH_ transistors and the active die area was 0.432 mm^2^. The temperature resolution was 0.49 °C and the temperature error was from −1.6 to +0.6 °C over the range of 0 to 100 °C after two-point calibration. The supply voltage sensitivity was 0.085 °C/mV. The conversion rate was 25kHz and the energy efficiency was 7.2 nJ/sample.

## 1. Introduction

Temperature sensors have been increasingly demanded in various applications such as military, aerospace, scientific research, industry, agriculture, medicine, transportation and so on. Based on the sensory device (CMOS, BJT or resistor) and the measured signal type (voltage, current, frequency, delay time, phase shift or bandwidth), they can be implemented in various ways [1,2,3,4,5,6,7,8,9,10,11,12,13,14,15,16,17,18,19,20,21,22]. In this paper, we have chosen a time domain CMOS temperature sensor by considering the following factors.

**Figure 1:**
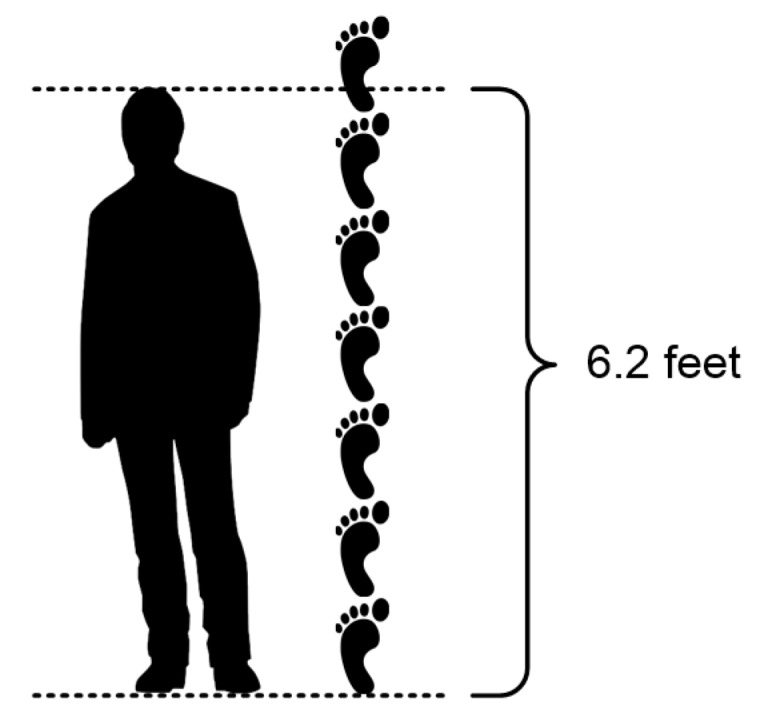
Measuring something is to represent it by a ratio of two quantities.

First, measuring something is to represent it by using a ratio of two quantities, i.e., one is to be measured and the other is to be a reference. For example, the height of a person can be represented by a multiple of foot length as shown in Figure 1. So, we need both the person and the reference foot length to measure the height. Even when the height is measured in a metric system, we also need both the person and the reference meter.

Second, measuring temperature by using a temperature sensor does not mean that we can directly measure temperature itself. What we can really measure by using a temperature sensor are the voltage or current signals or the frequencies or delay times which are one-to-one matched to the temperature.

Third, as CMOS process technology improves, the supply voltage is getting lower and lower and the clock speed is getting higher and higher, which means that the voltage resolution is degraded, whereas the time resolution is improved. This trend has naturally made time domain signal-based circuits more attractive than ever before.

**Figure 2:**
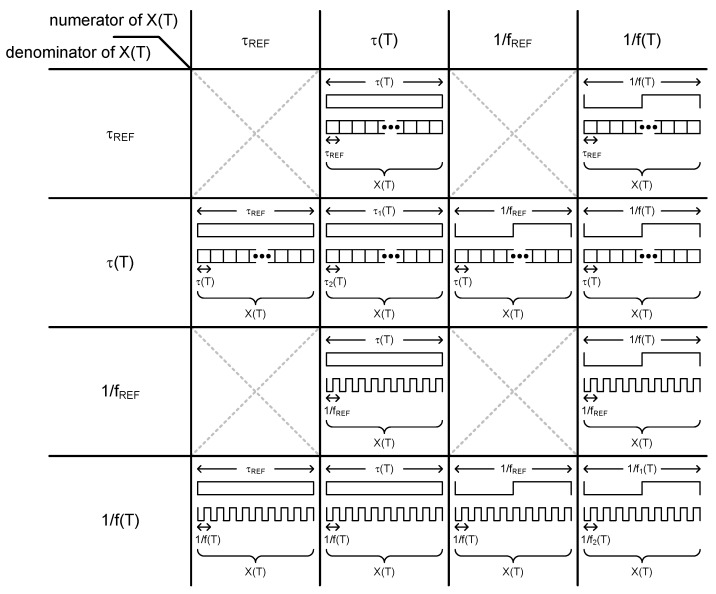
Twelve types of operational principles based on X(T).

Thus, in this paper we present a new type of time domain CMOS temperature sensor which can estimate temperature by measuring a frequency and a delay time. As summarized in Figure 2, a time domain temperature sensor can be implemented by choosing one among 12 types of operational principles. These operational principles have been categorized as shown in the figure based on the temperature estimation function, X(T), which is, in this paper, defined as the ratio of two quantities chosen from τ_REF_, τ(T), 1/f_REF_ and 1/f(T). Here, T is the temperature and τ_REF_ is defined as a temperature-independent reference delay time of a delay cell or a delay line, and τ(T) is defined as a temperature-dependent delay time of a delay cell or a delay line. Similarly, f_REF_ is defined as a temperature-independent reference frequency of an oscillator or a clock signal, and f(T) is defined as a temperature-dependent frequency of an oscillator or a clock signal. If we arbitrarily choose two quantities from these four different kinds of time domain signals and put them into the numerator and denominator of X(T), respectively, overall 16 types of operational principles can theoretically exist, as shown in Figure 2. However, if the two quantities are irrelevant to T at the same time, we cannot use them for temperature estimation.

**Figure 3:**
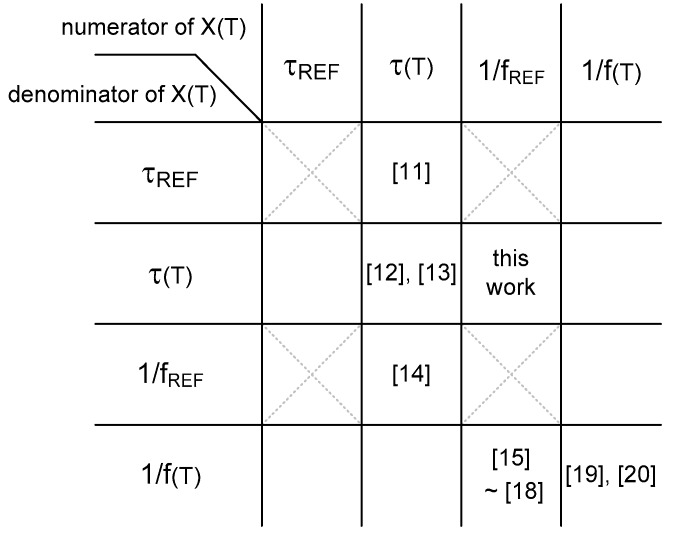
Prior works in time domain CMOS temperature sensors [11,12,13,14,15,16,17,18,19,20] and this work.

Accordingly, from the perspective of X(T), we can newly categorize the previous time domain CMOS temperature sensors, as shown in Figure 3. In [11], X(T) is defined as the temperature-dependent delay time, τ(T), of an open-loop delay line divided by the reference delay time, τ_REF_, of a delay cell in the reference delay locked loop (DLL). A finite state machine (FSM) is used to find out the quantized digital data which corresponds to X(T). In [12], X(T) is defined as the temperature-dependent delay time difference, τ_1_(T) − τ_REF_, of two delay lines divided by the other temperature-dependent delay time, τ_2_(T), generated from the cyclic time-to-digital converter (TDC). In [13], the temperature-dependent delay time, τ_1_(T), of the first delay line is divided by the other temperature-dependent delay time, τ_2_(T), of the fine delay cells in the second adjustable curvature-compensating reference delay line. A successive approximation register (SAR) control logic is used to obtain the quantized digital value of X(T). In [14], the temperature-dependent delay time difference, τ_1_(T) − τ_2_(T), of two delay lines is divided by the reference clock period, 1/f_REF_, of an external reference clock. In [15,16], the reference clock period, 1/f_REF_, of the temperature-independent oscillator is divided by the clock period, 1/f(T), of the temperature-dependent oscillator. In [17], the reference clock period, 1/f_REF_, of an external reference clock is divided by the clock period, 1/f(T), of the temperature-dependent oscillator. In [18], the reference clock period, 1/f_REF_, of an external reference clock is first divided by the clock period, 1/f_1_(T), of the temperature-dependent oscillator and then divided by the other clock period, 1/f_2_(T), of the linearity controlling oscillator, respectively. Then, X(T) is obtained by taking the difference between them, i.e., X(T) = 1/f_REF_/1/f_1_(T) − 1/f_REF_/1/f_2_(T). In [19,20], X(T) is defined as the first clock period, 1/f_1_(T), of the temperature-dependent oscillator divided by the second clock period, 1/f_2_(T), of the other temperature-dependent oscillator.

In addition to the aforementioned works shown in Figure 3, we can also design a different type of time domain CMOS temperature sensor based on the other operational principle. In this paper, we present a new type of time domain CMOS temperature sensor based on X(T) which is defined as X(T) = 1/f_REF_/τ(T), as shown in Figure 3. By choosing X(T) = 1/f_REF_/τ(T), we can obtain a relatively fast temperature conversion rate since the temperature sensor has τ(T) in the denominator of X(T) and does not need to count large numbers as do the other kinds of temperature sensors, which have 1/f(T) or 1/f_REF_ in the denominator of X(T). Compared to [11,12,13], this temperature sensor also does not need dual delay lines, since it has τ(T) only in the denominator of X(T).

This paper is organized as follows. In Section 2, we present the architecture and the operational principle of the implemented time domain CMOS temperature sensor. We also discuss some major aspects closely related to the performance of the temperature sensor. Section 3 explains the circuits of the main building blocks and Section 4 shows the measured results and compares the performance with the previous works. The conclusion is given in Section 5.

## 2. Architecture

**Figure 4:**
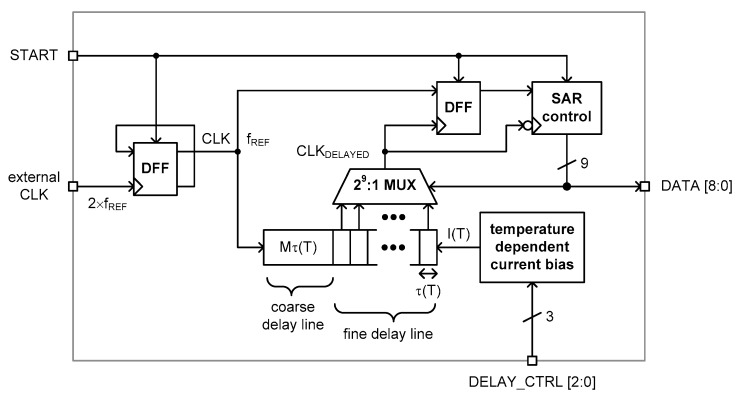
Architecture.

Figure 4 shows the architecture of the proposed time domain CMOS temperature sensor. This temperature sensor consists of a temperature-dependent current bias circuit, a coarse delay line, a fine delay line including 2^9^ fine delay cells, a 2^9^:1 MUX, a 9b SAR control logic and two DFFs.

The temperature-dependent current bias circuit generates a reference current, I(T), which is linear with the temperature. This current is fed to the coarse and fine delay lines to control the delay time of the delay lines. The delay time of the coarse delay line is designed to be about 80% of the reference clock period, 1/f_REF_, and the delay time of the fine delay line varies from 0% to about 40% of 1/f_REF_ depending on the number of fine delay cells selected by the 2^9^:1 MUX. By selecting one from 2^9^ delayed clock signals, the DFF can compare both the rising edges of the original clock signal, CLK, and the delayed clock signal, CLK_DELAYED_, and determine which one between CLK and CLK_DELAYED_ is faster than the other. Thus, the SAR control logic can update the 9b data, DATA [8 : 0], repeatedly until the total delay time of the coarse and fine delay lines equals the reference clock period, 1/f_REF_. After a complete sequence of SAR operations, Mτ(T) + X(T)τ(T) = 1/f_REF_ where τ(T) is the delay time of the fine delay cell, Mτ(T) is the delay time of the coarse delay line and X(T)τ(T) is the delay time of the fine delay line. This takes nine clock periods in total, i.e., 9/f_REF_, for the SAR control logic to finish a complete sequence of SAR operations. Since the 9b output data, DATA[8 : 0], is equivalent to the quantized digital value of X(T), X(T) can be represented as (1). This temperature sensor is activated by the rising edge of the external trigger signal, START.

(1) $$X(T)=\frac{\frac{1}{f_{REF}}}{\tau (T)}-M$$

Meanwhile, the performances of this temperature sensor were closely related to the following factors: temperature linearity, process variation compensation and supply voltage insensitivity.

**Figure 5:**
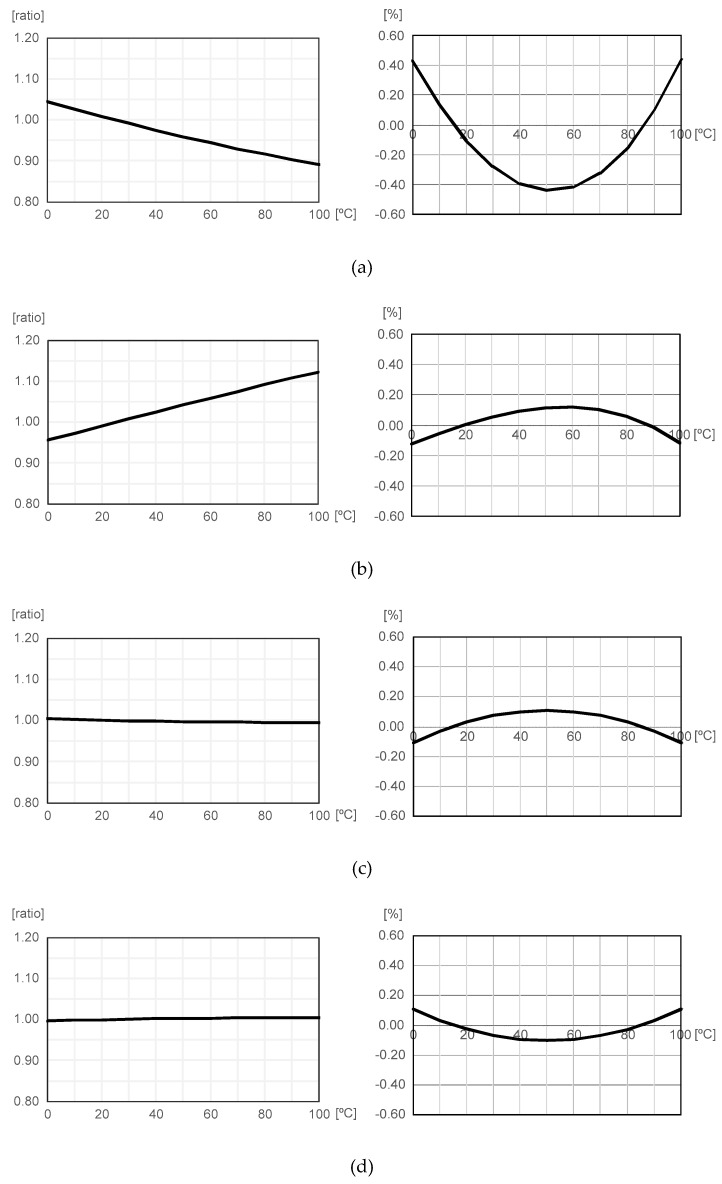
Temperature-dependent variation and linearity error of (**a**) R_N_(T), (**b**) 1/R_N_(T), (**c**) R_P_(T) and (**d**) 1/R_P_(T).

Having a good temperature linearity means that X(T) can be approximately expressed as X(T) = aT + b, where a and b are constants. If X(T) is linear with T, then we can accurately estimate temperature by simply measuring X(T) and by using linear interpolation. Thus, selecting the most appropriate one from various sensing elements available in the standard CMOS processes such as mobility, threshold voltage, resistance and so on, is important for good temperature linearity. Among them, mobility has relatively poor temperature linearity and threshold voltage is not easy to manipulate [11,12,13,14]. Since poly resistors have relatively good temperature linearity and are easy to manipulate, we simulated the temperature linearity of n-type and p-type poly resistors as summarized in Figure 5. In this figure, R_N_(T) and R_P_(T) are the resistances of n-type and p-type poly resistors, respectively. Here, the temperature-dependent variation was normalized with respect to the value measured at 25 °C and the linearity error was defined as the deviation (%) from the linear fit of the values of R(T) or 1/R(T) over the range of 0 to 100 °C. As a sensing element, we chose 1/R_N_(T) in this work because R_P_(T) and 1/R_P_(T) were useless because they were almost constant over the range of 0 to 100 °C as shown in Figure 5c,d. Between 1/R_N_(T) and R_N_(T), 1/R_N_(T) had better temperature linearity than R_N_(T), as shown in Figure 5a,b. Over the range of 0 °C to 100 °C, 1/R_N_(T) changed by up to 16% and had the low linearity error of ±0.12%, as shown in Figure 5b.

Since the process corner is determined during chip fabrication, the chip characteristic is time-invariant after chip-out. That is, once we know the values of a and b in X(T) = aT + b, we can use them repeatedly for temperature estimation thereafter. Thus, we needed to carry out two-point calibration to find out the values of a and b. Specially, only if X(T) = aT, or in other words, b = 0, one-point calibration may be used for process variation compensation.

The values of a and b are actually affected if the supply voltage is shifted from its nominal value. To suppress the supply voltage sensitivity, we can choose one of the following options. The first option is to use an additional regulator for the temperature sensor [2,17,21]. The second option is to carefully devise a supply voltage-insensitive architecture such that the values of a and b are independent of the supply voltage variation. For this purpose, we can make the numerator and the denominator of X(T) equally affected by the supply voltage variation or not affected by the supply voltage variation at all. In this paper, we tried to make the numerator and the denominator of X(T) be as little affected by the supply voltage variation as possible.

## 3. Circuit Implementation

### 3.1. Temperature-Dependent Current Bias Circuit

**Figure 6:**
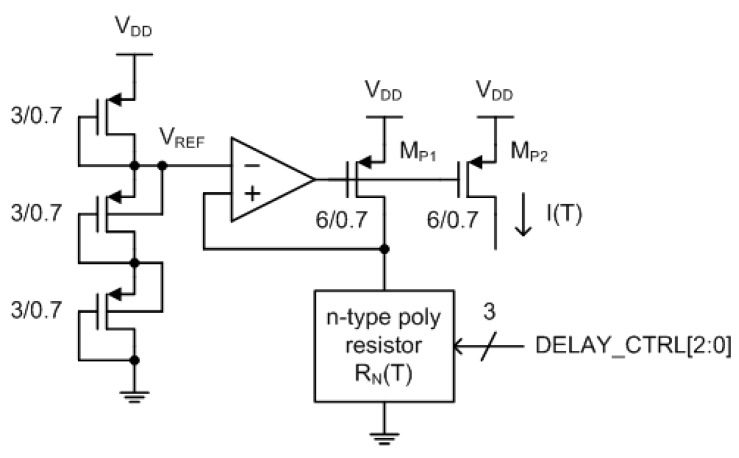
Temperature-dependent current bias circuit.

Figure 6 is the temperature-dependent current bias circuit implemented in a standard 0.18 μm 1P6M bulk CMOS technology with general V_TH_ transistors. This circuit generates a reference current, I(T), which is inversely proportional to the resistance of an n-type poly resistor, R_N_(T). By placing three diode-connected PMOS transistors in series between V_DD_ and 0V, we can obtain the reference voltage, V_REF_, which equals 2 × V_DD_/3. Since M_P1_ and M_P2_ are matched, the current-mirrored reference current can be expressed as follows:

(2) $$I(T)=\frac{V_{REF}}{R_{N}(T)}=\frac{\frac{2}{3}V_{DD}}{R_{N}(T)}$$

**Figure 7:**
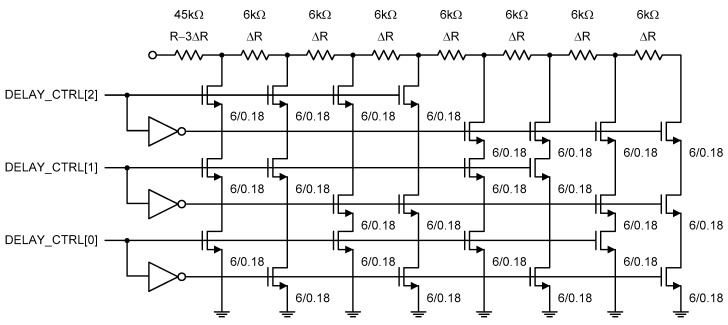
Digitally tuned n-type poly resistor.

Figure 7 shows the n-type poly resistor of the temperature-dependent current bias circuit. It can be tuned by the external 3b digital control word, DELAY_CTRL[2 : 0]. We implemented this digitally tunable n-type poly resistor array to roughly compensate for the probable severe process variation of the n-type poly resistor. If the resistance is shifted too far from its nominal value in the worst case corner, we cannot expect constant power consumption for this temperature sensor. Thus, the rough resistance tuning process should be carried out before two-point calibration.

### 3.2. Fine Delay Cell

**Figure 8:**
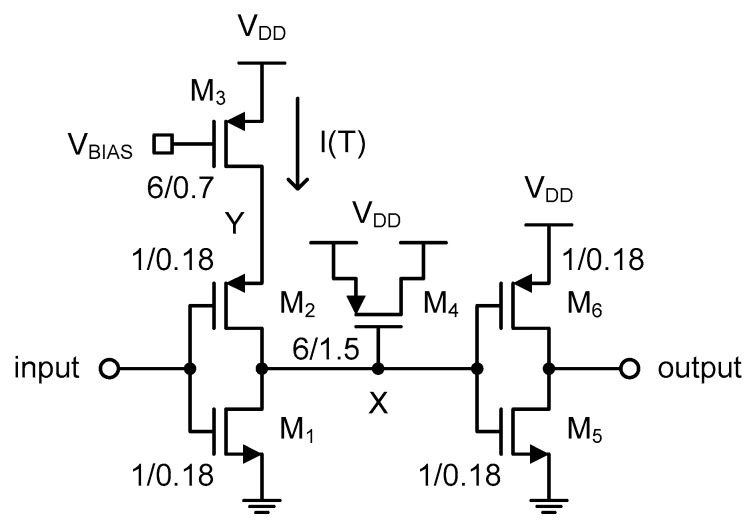
Fine delay cell.

Figure 8 shows the schematic of the fine delay cell. It is composed of a current-starved inverter followed by a PMOS capacitor and a normal inverter. The current-starved inverter has only a PMOS current source, such that the low to high propagation delay time of the delayed clock signal at node X was determined by the reference current, I(T), and the total capacitance, C, seen at node X. Contrarily, the high to low propagation delay time is very small and not affected by I(T) at all. The total capacitance, C, at node X is the sum of all kinds of parasitic capacitances of M_1_ ~ M_6_, in addition to the MOS gate capacitance of M_4_.

**Figure 9:**
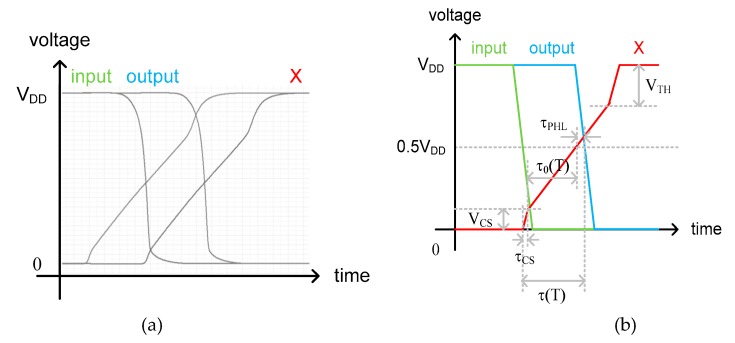
(**a**) Simulated and (**b**) theoretical waveforms of the fine delay cell.

**Figure 10:**
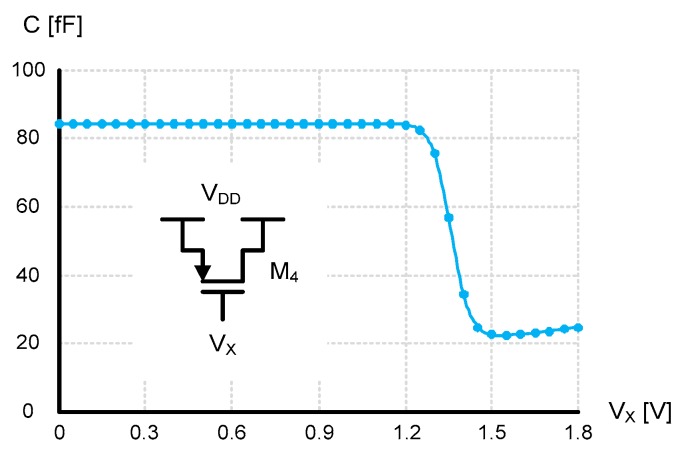
Capacitance versus voltage curve of the PMOS capacitor, M_4_.

Figure 9 shows the simulated and theoretical waveforms of the input and output clock signals of the fine delay cell. The delayed clock signal at node X is also denoted in red. As shown in the figure, the delayed clock signal at node X goes through three different slope regions when rising up from 0V to V_DD_. The first slope is related to the charge sharing between node X and node Y of Figure 8 right after M_2_ is turned on. When the input clock signal is initially high, as shown in Figure 9b, M_2_ and M_3_ are both turned off, and so the voltage at node Y is charged to V_DD_. When M_2_ is turned on by the falling edge of the input clock signal, the charge stored at node Y is instantly shared with node X right before I(T) of M_3_ starts charging the total capacitance, C, at node X. Thus, the second slope is determined by I(T) and C. The delay time, τ_0_(T), in this region can be expressed as

(3) $$\tau_{0}(T)=\left(\frac{V_{DD}}{2}-V_{CS}\right)\times \frac{C}{I(T)}$$

where V_CS_ is the charge-shared voltage and τ_CS_ is the short delay time during charge sharing. Finally, the third slope differs from the second slope and shows up when *V_SG_* of M_4_ decreases to be smaller than the threshold voltage of M_4_, and so the PMOS gate oxide capacitance is abruptly reduced, as shown in Figure 10. Since the total capacitance, C, at node X is reduced while I(T) is kept constant, the slope steepens correspondingly. To prevent the low to high propagation delay time of the delayed clock signal at node X from being affected by this third slope region, we used the PMOS capacitor rather than the NMOS capacitor, as shown in Figure 8. Additionally, τ_PHL_ is the high to low propagation delay time of the output clock signal. Thus, the delay time, τ(T), of the fine delay cell can be represented as

(4) $$\tau (T)=\tau_{CS}+\tau_{0}(T)+\tau_{PHL}=\tau_{CS}+\left(\frac{V_{DD}}{2}-V_{CS}\right)\times \frac{C}{I(T)}+\tau_{PHL}$$

For simplicity of analysis, we assume that τ_CS_ and τ_PHL_ are much smaller than τ_0_(T), and V_CS_ is also much smaller than V_DD_, respectively. Then, the delay time of the fine delay cell can be approximately represented as

(5) $$\tau (T)\approx \frac{V_{DD}C}{2I(T)}.$$

Now, by substituting (2) and (5) into (1), we can obtain the X(T) of this temperature sensor as follows:

(6) $$X(T)=\frac{4}{3f_{REF}R_{N}(T)C}-M$$

In Section 2, we chose 1/R_N_(T) as the sensing element of this temperature sensor. Now we can see that X(T) is linear with 1/R_N_(T) as expected.

### 3.3. SAR Control Logic

In this temperature sensor, the SAR control logic updates the 9b data, DATA[8 : 0], periodically at the falling edge of CLK_DELAYED_. It sends DATA[8 : 0] to the 2^9^ : 1 MUX to select the most appropriate one from 2^9^ delayed clock signals until the delay time of the coarse and fine delay lines equals to 1/f_REF_.

**Figure 11:**
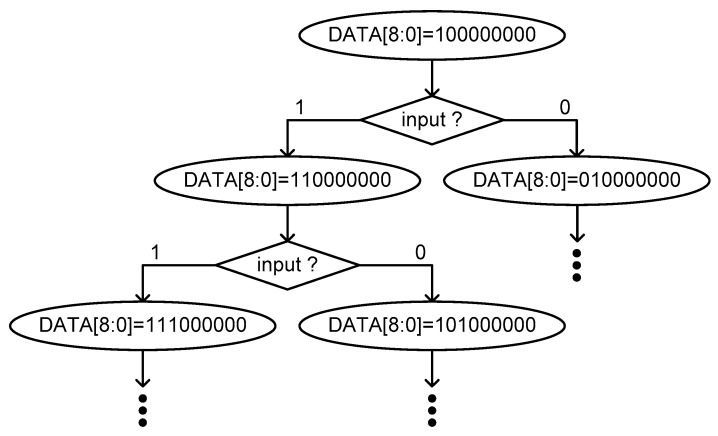
SAR state diagram.

Figure 11 shows the state diagram of the implemented SAR control logic. First, it starts from the 9b data, DATA[8 : 0], initialized as 100000000. This initial code points at the center of 2^9^ fine delay cells of the fine delay line. When both rising edges of CLK and CLK_DELAYED_ are compared by the DFF, as shown in Figure 4, it outputs 1 or 0 as a comparison result to the input of the SAR control logic. If the value is 1, it means that the rising edge of CLK is faster than the rising edge of CLK_DELAYED_ and if the value is 0, vice versa. Depending on the input of the SAR control logic, DATA[8 : 0] is next updated to 110000000 or 010000000 and the 2^9^:1 MUX selects a faster or slower one from 2^9^ delayed clock signals, as shown in Figure 11. After nine clock periods, i.e., 9/f_REF_, the SAR control logic finally outputs the 9b digital data as DATA[8 : 0].

**Figure 12:**
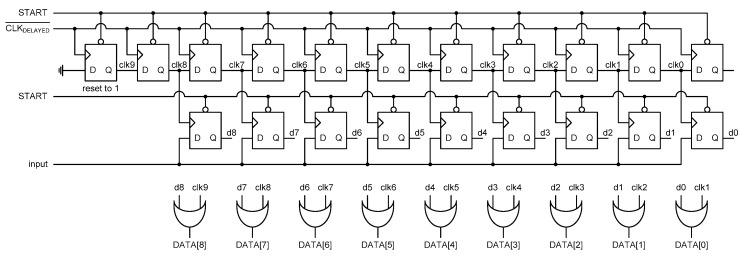
SAR control logic.

Figure 12 shows the implemented SAR control logic. It consists of 20 DFFs and nine OR gates. Among the 20 DFFs, 11 DFFs on the upper side are used as the clock generator and nine DFFs on the lower side are used as the shift register to store the input value sequentially. Since an impulse clock signal like 010 is needed by only one of the nine DFFs at a time in the shift register, the leftmost DFF of the clock generator was designed as a different type of DFF which is reset to 1 rather than 0. All the other DFFs of the SAR control logic are reset to 0 when the START signal is low.

## 4. Measurements

**Figure 13:**
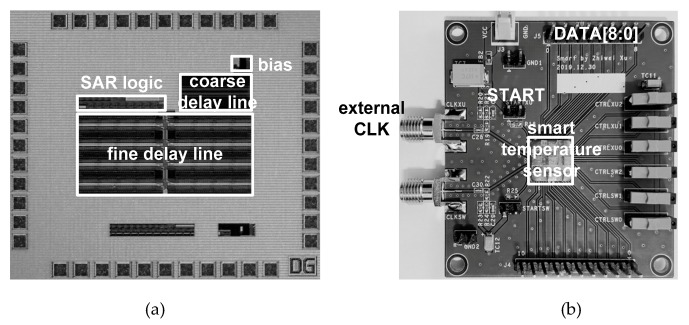
(**a**) Die photo and (**b**) FR4 PCB.

The proposed temperature sensor was implemented in a standard 0.18 μm 1P6M bulk CMOS process with general V_TH_ transistors. Figure 13 shows the die photo and the FR4 PCB. The active die area was 0.432 mm^2^. It occupied a relatively large area because the fine delay line contained as many as 2^9^ fine delay cells for a temperature resolution as low as 0.49 °C while covering up more than 0 to 100 °C of the temperature range with margins. Thus, the die area can be reduced if the temperature resolution and temperature range requirements are mitigated.

**Figure 14:**
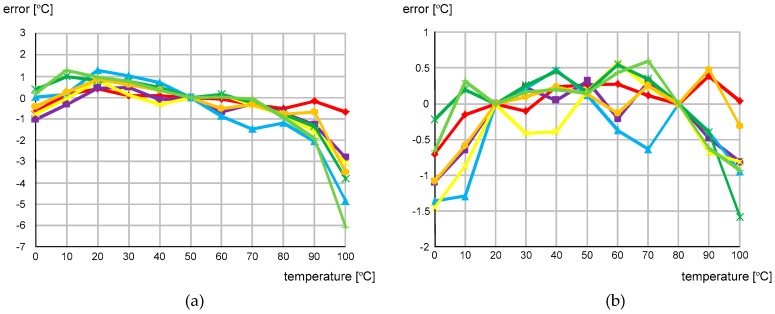
Measured temperature error after (**a**) one-point calibration and (**b**) two-point calibration.

**Figure 15:**
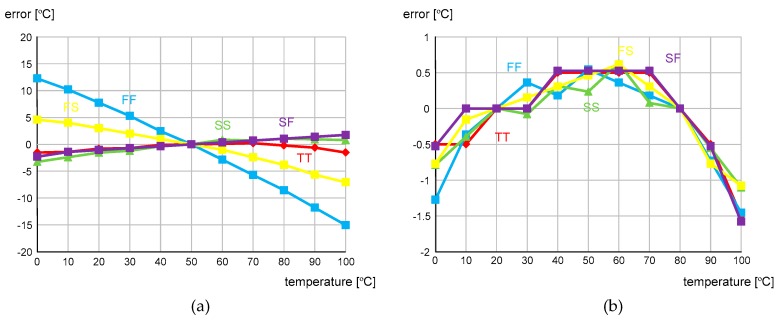
Simulated temperature error after (**a**) one-point calibration and (**b**) two-point calibration for the process corners, TT, FS, SF, FF and SS.

Figure 14 shows the measured temperature error over the range of 0 to 100 °C after one-point and two-point calibrations, respectively. The temperature error was measured with seven different temperature sensor chips in the temperature chamber, JEIO TC3-ME-025. For measurement accuracy, a PT100 RTD sensor (OMEGA PR-20 with DP32PT) was used as a reference. The measured temperature error varied from −6.1 to + 1.3 °C after one-point calibration at 50 °C and from −1.6 to +0.6 °C after two-point calibration at 20 and 80 °C. Figure 15 shows the simulated temperature error after one-point and two-point calibrations, respectively. For the process corners, TT, FS, SF, FF and SS, the simulated temperature error varied from −15.0 to + 12.3 °C after one-point calibration at 50 °C and from −1.6 to +0.6 °C after two-point calibration at 20 and 80 °C. This result agrees well with the derived Equation (6) in that X(T) is represented as aT + b and thus, we needed two-point calibration to compensate for the process variations in a and b.

**Figure 16:**
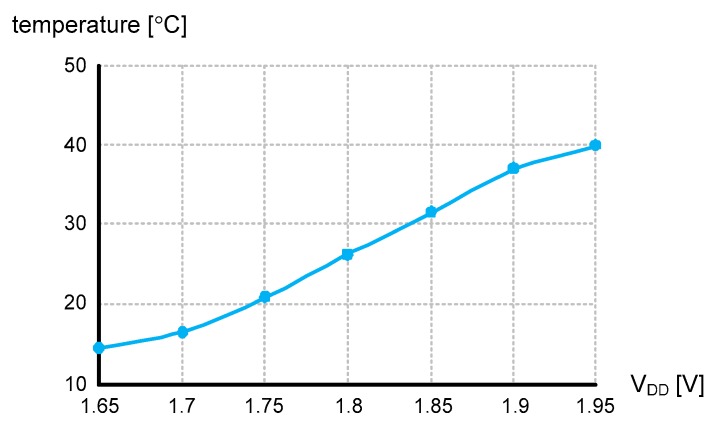
Measured V_DD_ sensitivity.

The supply voltage sensitivity was measured as low as 0.085 °C/mV at 27 °C while V_DD_ varied from 1.65 to 1.95 V, as shown in Figure 16. From (6), X(T) is ideally independent of V_DD_ because it is not a function of V_DD_ at all. However, the X(T) of (6) was derived under the assumption that τ_CS_ and τ_PHL_ are much smaller than τ_0_(T), and V_CS_ is also much smaller than V_DD_, respectively. Since τ_CS_, τ_PHL_ and V_CS_ are very small but not zero, X(T) is slightly affected by V_DD_. To reduce the supply voltage sensitivity, we canceled out V_DD_ in (5) by carefully designing I(T) to also be linear with V_DD_, as shown in (2).

**Figure 17:**
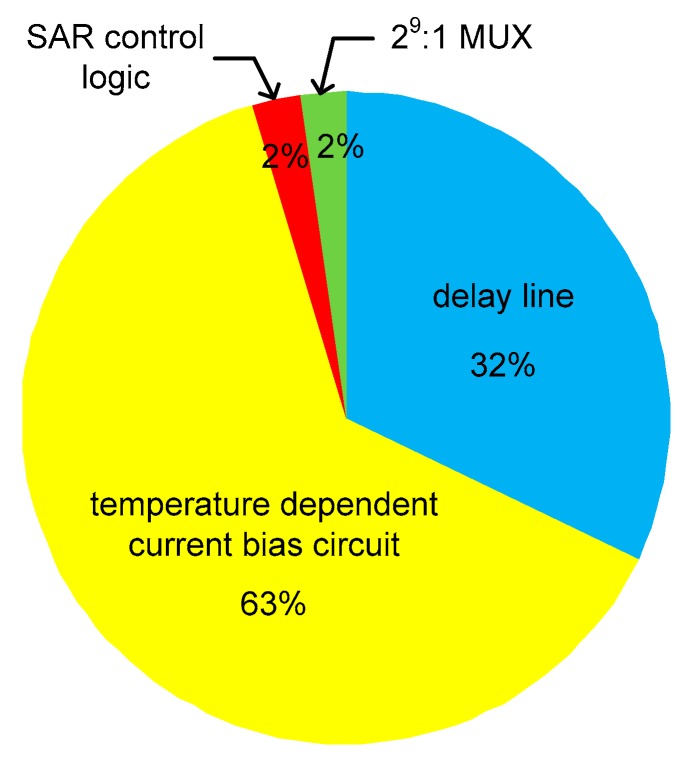
Power consumption breakdown.

The reference clock frequency, f_REF_, was 225 kHz. Since the temperature sensor operates based on the 9b SAR control logic, the conversion rate was 25 kHz and the energy efficiency was 7.2 nJ/sample. Figure 17 shows the power consumption breakdown at 25 °C. Sixty-three percent of the total power was consumed by the temperature-dependent current bias circuit and 32% was consumed by the coarse and fine delay lines. Table 1 compares the implemented temperature sensor with the previous time domain CMOS temperature sensors implemented in 0.13 μm or 0.18μm CMOS technologies. Compared to previous works, this temperature sensor had a relatively fast conversion rate.

## 5. Conclusions

In this paper, we defined the temperature estimation function, X(T), and categorized the time domain CMOS temperature sensors into 12 types. By choosing X(T) = 1/f_REF_/τ(T), we implemented a new type of time domain CMOS temperature sensor with a 9b SAR control logic and a fine delay line. Although the chip has a relatively large die area, it can achieve a comparable temperature resolution and linearity error. The supply voltage sensitivity could be fairly reduced by carefully designing I(T) to cancel out V_DD_ in τ(T).

## Figures and Tables

**Table 1:** Performance summary.

	[11]	[14]	[16]	[20]	[21]	This Work
CMOS technology	0.13 μm	0.18 μm	0.18 μm	0.18 μm	0.18 μm	0.18 μm
Die area	0.12 mm^2^	0.0324 mm^2^	0.09 mm^2^	0.074 mm^2^	0.19 mm^2^	0.432 mm^2^
Supply voltage	1.2 V	1.0 V	1.2 V	0.8 V	1.2 V	1.8 V
Temperature range	0~100 °C	0~100 °C	0~100 °C	−20~80 °C	−40~85 °C	0~100 °C
Resolution	0.78 °C	0.3 °C	0.3 °C	0.145 °C	0.18 °C	0.49 °C
Accuracy	−4.0~4.0 °C	−0.8~1.0 °C	−1.4~1.5 °C	−0.9~1.2 °C	−1.0~1.0 °C	−1.6~0.6 °C
Conversion rate	5 kHz	1 kHz	33.3 Hz	1.2 Hz	1kHz	25 kHz
Energy	0.24 μJ/sample	0.41 nJ/sample	2.2 nJ/sample	8.9 nJ/sample	−	7.2 nJ/sample
V_DD_ sensitivity	1.6 °C/mV	−	0.014 °C/mV	0.0038 °C/mV	−	0.085 °C/mV
